# Tentorial Notch Meningiomas: Innovative Preoperative Management and Literature Review

**DOI:** 10.3389/fsurg.2022.840271

**Published:** 2022-03-09

**Authors:** Delia Cannizzaro, Davide Milani, Ismail Zaed, Maria Pia Tropeano, Federico Nicolosi, Francesco Costa, Franco Servadei, Maurizio Fornari, Vincenzo D'Angelo, Andrea Cardia

**Affiliations:** ^1^Department of Neurosurgery, Humanitas Clinical and Research Center – IRCCS, Rozzano, Italy; ^2^Department of Neurosurgery, Hospital “Casa Sollievo Della Sofferenza”, San Giovanni Rotondo, Italy

**Keywords:** intracranial meningioma, tentorial notch, incisural meningioma, presurgical planning, neuronavigation

## Abstract

**Background:**

Tentorial meningiomas account for only 3–6% of all intracranial meningiomas. Among them, tentorial incisura (notch) location must be considered as a subgroup with specific surgical anatomy and indications, morbidity, and mortality. In this study, we propose an update on preoperative management in order to reduce postoperative deficits.

**Methods:**

We retrospectively collected adult patients treated for incisural meningioma between January 1992 and December 2016 in two different neurosurgical departments. Demographic, clinical, and neuroradiological preoperative and postoperative data were analyzed. In the most recent subgroup of tumors, a preoperative digital simulation was performed to define a volumetric digital quantification of the tumor resection. A review of the pertinent literature has been also done.

**Results:**

We included 26 patients. The median age was 58.4 years. Onset neurological signs were cranial nerve deficit in 9 patients, hemiparesis in 7, gait disturbance in 3, and intracranial hypertension in 3 patients. Simpson grade I removal was achieved in 12 patients, II in 10, III in 3, and IV in 1 patient. An overall rate of 23% postoperative complications was observed. The average follow-up duration was 68.5 months. Residual tumor was reported in 8 patients. Five patients underwent gamma knife radiosurgery. In 34.6% of patients, the surgical approach was chosen with preoperative digital planning estimating the potential volume of postoperative residual tumor, the target for radiosurgical treatment.

**Conclusions:**

A multidisciplinary approach to plan incisural meningiomas management is important. To lower postoperative morbidity and mortality, a careful preoperative case analysis is useful. A planned residual tumor, supported by preoperative simulation imaging, could be safely treated with radiosurgery.

## Introduction

Meningiomas are the most frequent intracranial benign lesions. Intracranial meningiomas are classified as dural-based tumors, due to their origin from the dura mater layer. Therefore, they can be localized in any cranial district where the dura mater is present (1).

Among the different types of meningiomas, those located in the tentorium are still a topic of discussion because of the difficulty of their management. Among all meningiomas, tentorial meningiomas constitute the 3–6% of all intracranial meningiomas (2, 3). Among tentorial meningiomas, we focus our attention on meningiomas of tentorial incisura, defined according to Yasargil classification (4, 5). Tentorial incisura or tentorial notch is the unique portion of tentorium that is not close to the skull base, it can be divided into anterior, lateral or medial, and posterior space. Meningiomas of this area are generally lateral or posterior (6).

The current neurosurgical gold standard treatment is based on careful balancing between radical resection and suitable neurological functioning. Therefore, the demonstrated effectiveness of alternative treatment, such as radiosurgery and radiotherapy, has progressively changed the surgical treatment goal. Preserving a good quality of life has to be encouraged compared to radical unsafe resection.

We present our experience in the management of tentorial notch meningiomas focusing on T1 and T2 incisural meningiomas. We analyzed indications, preplanning studies, neuroimaging findings, surgical technique, and clinical/radiological results. We performed a pertinent literature review to validate our results. Moreover, in our study, we describe a novel workflow for the digital simulation and 3D reconstruction. This virtual technique allows a digital prediction of tumor removal extent.

## Materials and Methods

We performed a retrospective observational study collecting adult patients who underwent a neurosurgical procedure for intracranial tentorial meningioma between January 1992 and December 2016 in two different Italian neurosurgical departments (Hospital “Casa Sollievo della Sofferenza” and Humanitas Research Hospital).

The electronic databases have been searched for intracranial-meningioma patients with histological confirmation by 2 different neurosurgeons. Radiological images of MRI were analyzed to extract tentorial notch meningiomas according to Yasargil classification (5). Tentorial meningiomas that were not T1-T2 incisural meningiomas, according to 1996 Yasargil's classification of tentorial meningiomas, were excluded. Meningiomas with secondary involvement of the tentorium were excluded.

### Demographic and Surgical Data

For patients included in our study, we considered the demographical and clinical data, namely, preoperative clinical and neurological status, intra- and postoperative complications, and postoperative clinical and neurological disorders, histological grade. All this information has been extracted by the authors from the electronic medical records of the institutions.

Experienced neuroradiologists evaluated the tumor size as the maximal diameter on contrast-enhanced MRI in preoperative and postoperative images. Simpson grade-classification system was used to demarcate tumor resection.

### Preoperative Radiological Assessment

A preoperative MRI examination was performed that included T1-weighted imaging, T2-weighted imaging, and T1-weighted imaging with contrast enhancement (administered with gadolinium) for all patients.

Cerebral angiography was performed in carefully selected cases: tumors size >30 mm or complex involvement of vascular structures.

### Virtual Simulation

We selected a subgroup of the latest 6 patients for a preoperative digital simulation in order to define volumetric digital quantification of the tumor resection vs. tumor residual. The software achieves a realistic 3D reconstruction of the tumors and of the brain with a sensible reproduction of surgical brain retraction. Further prospective analysis has been performed only on 6 patients, since it was not available before.

### Digital Imaging and Communications in Medicine (DICOM) Data Acquisition and Registration

The radiological datasets used for the digital simulations were 3D CT, 3D MP-Rage (Three Dimensional Magnetization Prepared Rapid Acquisition GRE) MRI, T1-weighted MRI, Fiesta (Fast Imaging Employing Steady-state Acquisition) C/CISS MRI, DTI, and V-Phase Angio-MRI.

CT imaging data were acquired on Siemens Plus 4 Volume Zoom scanner (Siemens Medical Solutions, Inc., Malvern, PA, USA) according to a standard 3D CT protocol (0.4 mm slice thickness; 0-degree angulation; 100 ml of Omnipaque 350 [GE Healthcare, Inc., Princeton, NJ, USA] at 3 ml/s; 15-second scan delay). The sequence was transferred into DICOM format to a computer workstation (ASUS DX890, ASUSTeK Computer Inc., Taipei, Taiwan, Ram 2.4 Ghz, processor Intel i7) for analysis.

MR images were acquired using a 3T system (Verio, Siemens AG, Munich, Germany). Heavily T2-weighted images were obtained by 3D CISS sequence with the following parameters: TR 10.15 ms, TE 4.48 ms, flip angle 70°, fov 200 mm, matrix 512 × 384, slice thickness 0.80 mm, 1 acquisition. Magnetic resonance angiography was performed using a 3D time-of-flight sequence with the following parameters: TR 26.0 ms, TE 7.0 ms, flip angle 20°, fov 200 mm, matrix 320 × 320, slice thickness 0.90 mm, 1 acquisition. All the sequences were imported from the scanner using a DICOM receiver. The brain sequences were converted from DICOM (multi-file image storage) into NifTI (single-file image storage) format by using the DCM2NII module of MRIcron (Open-source software, http://people.cas.sc.edu/rorden/mricron/index.html).

To perform CT and MRI registration, we used a postoperative 3D CT dataset and a preoperative 3D MP-Rage or T1-weighted-contrasted dataset for all cases. Manual/automatic registrations have been obtained by using FMRIB's Linear Image Registration (FLIRT) of FSL and General Registration (Brain) tool of 3D Slicer (Open source—https://www.slicer.org).

### 3D Segmentation

**Figure 1:**
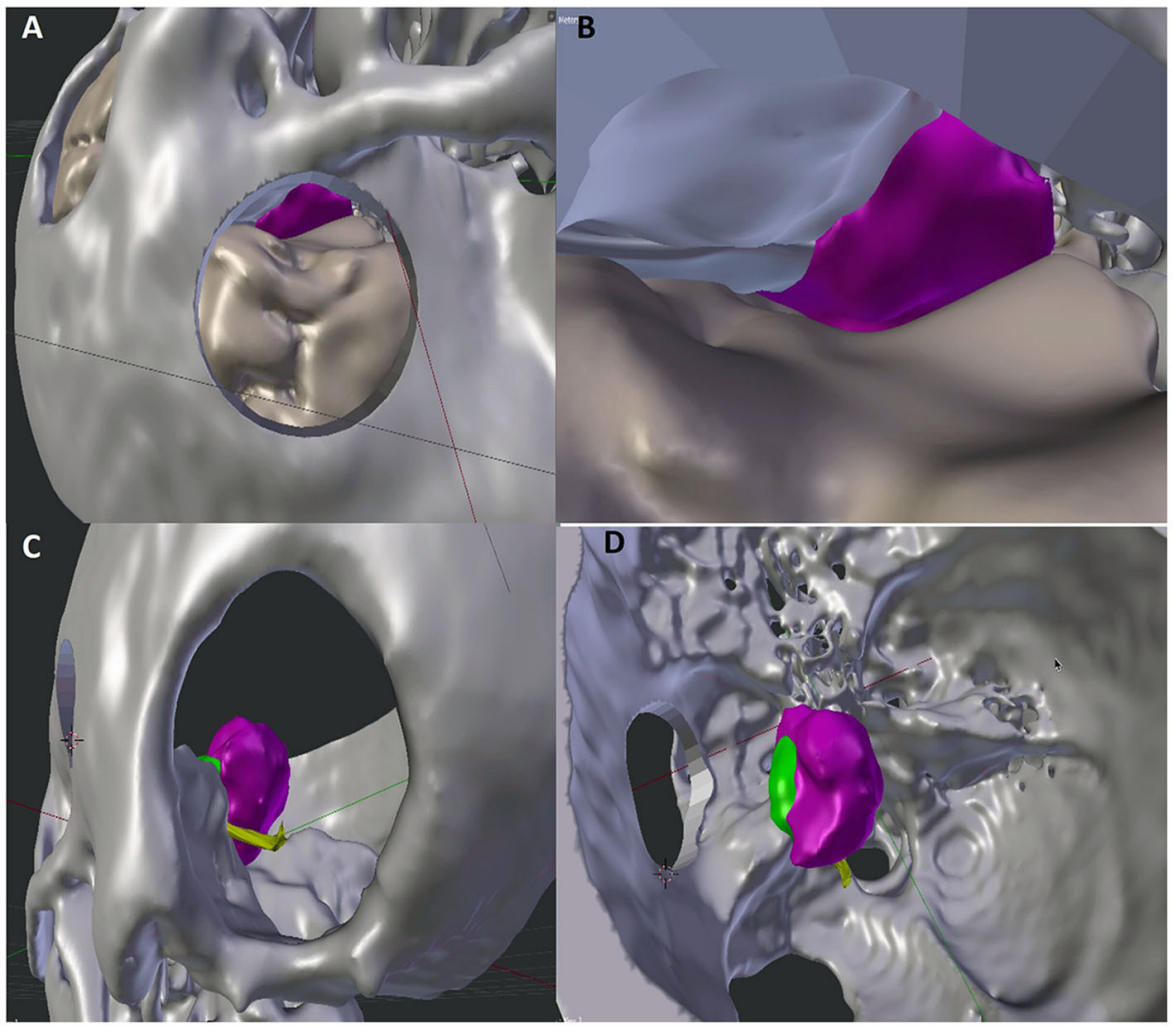
Comparison of 3D visualization of the lesion by subtemporal **(A,B)** and retrosigmoid **(C,D)** approaches. The improved visualization of neurovascular structures in the retrosigmoid approach reproduction allows a more accurate prediction of the expected residual (green portion).

The skull bone 3D segmentation was performed using the Gray Scale Model Maker tool of 3D Slicer, an intensity-based tool that was applied to bone window CT images in.dcm or.nii format. Postsegmentation cuts and operculum removal were manually done using 3D modeling tools, such as Blender (Open Source Software, Blender Foundation—Netherlands, https://www.blender.org) and Meshmixer (Autodesk, San Rafael, CA, USA, http://www.autodesk.com). An exemplificative case is shown in Figure 1.

Before proceeding with brain segmentation, a brain extraction was performed removing non-brain tissues, such as skin, fat, skull, and dura mater, using Brain Extraction Tool (BET), an automatic module of the FSL platform (free software, Oxford Center for Functional MRI of the Brain—Oxford, UK, https://fsl.fmrib.ox.ac.uk/fsl/fslwiki). FSL native format is NIfTI, therefore a prior DICOM to NIfTI conversion was done using DCM2NII script of MRIcron (open-source software, http://people.cas.sc.edu/rorden/mricron/index.html). After the extraction, brain MP-Rage MR sequences were segmented using Freesurfer (free software, Laboratory for Computational Neuroimaging Athinoula A. Martinos Center for Biomedical Imaging—Harvard Medical School—Boston, MA, USA, http://surfer.nmr.mgh.harvard.edu).

For the segmentation of cerebellum, brainstem, cranial nerves, supratentorial, and infratentorial tumors, we selected MP-Rage and T1 weighted datasets for brainstem, cerebellum and tumors, and C/CISS MR sequence for cranial nerves. We used manual segmentation tools, such as Editor/Model Maker of 3D Slicer and FMRIB's Automated Segmentation Tool (FAST) of FSL.

### Virtual Simulation of Tumor Resection

Once the anatomical segmentations were completed, a 3D scene was arranged using Blender. Two alternative craniotomies were made using Boolean functions. At this point, an excision of the tumor from each craniotomy was performed using Voxel (UpSurgeOn S.r.l., Milan). The software calculates the tumor resection rate that can be reached from the corresponding surgical window, considering a linear point view axis from an extracranial camera simulating the microscope camera. Two hypothetical resections were performed, one for each craniotomy, and the residual volume was quantified.

### Clinical and Radiological Follow-Up

Periodical clinical and neurological evaluations were performed subsequently the discharge at 1–6, 12–24, and 48 months after surgical procedures. Clinical follow-up was eventually modified according to the neurological condition varying of the patients. The clinical outcome was assessed with modified Rankin Scale (mRS) and assessed at 48 months of follow-up. The neuroradiological examination consisted of brain CT and/or MRI.

### Informed Consent

Written informed consent was reached in agreement with the Declaration of Helsinki. The study (retrospective analysis of electronic database) was approved by the Institutional Review Board (IRB) of the Humanitas Research Hospital (Reference no: IRB- 1459). For 3D virtual simulation, approval from the ethics committee was not required. Three dimensional reconstruction was applied for preliminary purposes and to non-decisional support of the surgical practice. The final decision on the best surgical approach was the result of multidisciplinary evaluations and defined by the senior author.

### Literature Review

In order to give a wider view of the therapeutic management of such lesions, a review of the literature has been performed. The search was performed on 2 different databases (PubMed and Google Scholar) and was conducted up to November 2021. In order to select all related articles, we selected general keywords: “tentorial meningioma” and “Tentorial notch meningiomas.” We included only case series in English, excluding all editorials, reviews of the literature, letters, and all articles written in other languages.

### Statistical Analysis

Outcomes and data of the descriptive statistics were reported with numbers, percentages, and median with range.

## Results

**Figure 2:**
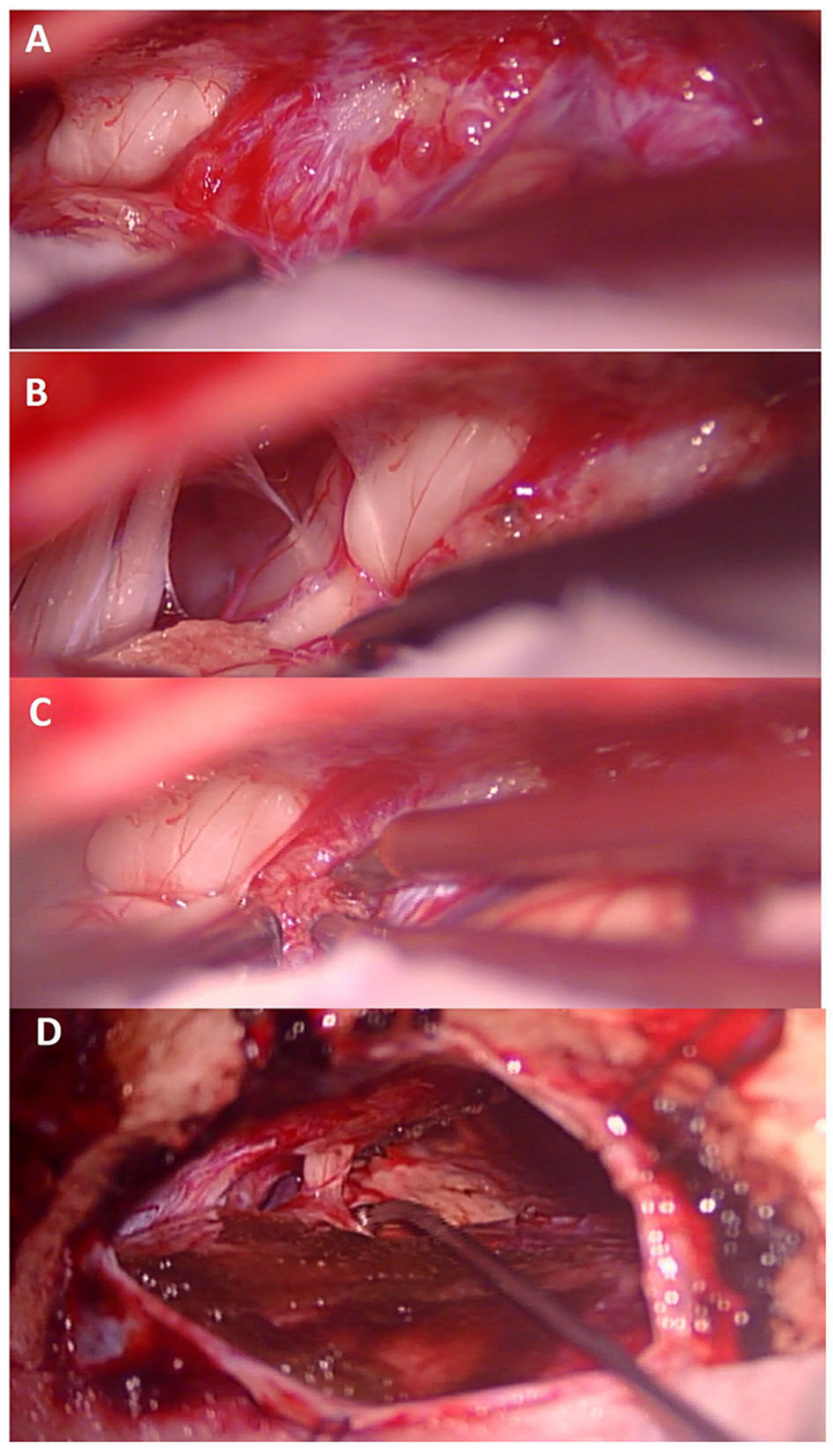
**(A)** After dural opening view of the tumor in relation to the seventh cranial nerve. **(B)** exposure of the lower cranial nerves and the seventh cranial nerve after partial removal of the tumor. **(C)** Lesional portion superior to the seventh cranial nerve. **(D)** Final view after removal of the meningioma showing the integrity of the seventh cranial nerve.

Between January 1992 and December 2016, a total of 93 patients with tentorial meningiomas underwent to the neurosurgical procedure has been identified in the centers involved in the study. Among the initial 93 cases, a total of 26 patients have been identified through radiological examinations and pathological records as a having tentorial notch or tentorial incisura (TNM) (T1 and T2; Figure 2).

### Demographic and Clinical Data

Demographic data are summarized in Table 1. The median age was 58.4 ± 6.7 years. We identified 17 women (65.3%) and 9 men (34.6%).

**Table 1:** Demographical and clinical data.

**Variable**	**Present series (*n* = 26)**	**T1 (12)**	**T2 (14)**
**Sex**, ***n*** **(%)**			
Male	9 (34.6)	4	5
Female	17 (65.3)	8	9
**Age, yrs**			
Median	58.4± 6.7	60.3	57.4
**Preoperative deficit**			
CN deficits	9(34.6%)	6	3
Hemiparesis	7 (26.9%)	1	6
Gait disturbance	3 (11.5%)	1	2
Intracranial hypertension	3 (11.5%)	2	1
Incidental	4	3	1
**Preoperative symptoms**			
Headache	20	11	9
Vertigo	15	7	8
Vomiting	8	4	4

We reported 22 (84.6%) symptomatic patients. The incidental discovery was achieved in 4 (15.3%) patients. The most frequent symptoms were headache (76.9%), vertigo (57.6%), and vomiting (30.8%). The cranial deficit was the most relevant onset sign, it compares in 9 cases (34.6%), 5 third cranial nerve, 2 fifth cranial nerve palsy, 2 forth cranial nerve palsy. Followed by hemiparesis in 7 patients (26.9%), gait disturbance 3 (11.5%), intracranial hypertension in 3 patients (11.5%), we proposed a surgical removal of the lesion in case tumor volume increases at radiological follow-up or in case of disabling symptomatology.

### Radiological Assessment

All patients underwent a preoperative contrast-enhanced MRI with neuronavigation sequences. Angiography was performed in 15 patients (57.69%).

### Anatomical and Surgical Data

The anatomical and surgical data are reported in Table 2. Among all the patients, 12 resulted to be T1 (46.2%), while the remaining 14 (53.8%) were classified as T2. The tumor diameter ranged for incisural meningioma from 26 to 48 mm. The median tumor size for T1 was 32 mm and 35 mm for T2. With an overall mean tumor size of 33.6, 1 patient (61.5%) was more than 30 mm.

**Table 2:** Summary of the surgical data.

**Variable**	**Present series (*n* = 26)**	**T1 (12)**	**T2 (14)**
**Median tumor size**			
	33.6 mm	32 mm	35 mm
**Tumor concistence**			
Soft	7	4	3
Tenacious	12	7	5
Solid	7	3	4
**Tumor removal**			
Simpson I	12 (46.1%)	5	6
Simpson II	10 (38.4%)	3	5
Simpson III	3(11.5%)	3	1
Simpson IV	1 (3.8%)	1	2
**Post oprative complication**			
Brain edema	1		1
Intracerebral hemorrhage	1		1
Mesencephalon infarction	1		1
Third cranial nerve deficit	3	2	1
**Mortality**	1		1
**Follow-up duration**			
Mean	68.5 months		
Range	14–308 months		
Recurrence/progression	8 (30.7%)		

In addition, the consistency of the lesion has been reported. Soft/crisp tumor consistencies were observed in 7 (26.9%), tenacious in 12 (46.1%), and solid in 7 (26.9%) cases. High arterial blood flow was copious in 18 cases (69.2%) and modest in 8 cases (30.7%) with the highest blood loss during surgery of 1,600 ml.

Meningioma resection was defined according to the Simpson classification; in particular, 12 patients (46.1%) were classified as Simpson grade I, 10 (38.4%) were included in the group of Simpson grade II, 3 were (11.5%) Simpson grade III patients, and 1 (3.8%) was Simpson IV in 1 (3.8%). Histopathological examination revealed anaplastic meningioma in 2 patients (7.7%). Intraoperative neurophysiological monitoring was used in all cases.

### Postoperative Complications

For T2 incisural meningiomas, the surgical complications were brain edema, intracerebral hemorrhage, and mesencephalon infarction in 3 different patients. We observed third nerve cranial permanent deficit in 1 patient. For T1 incisural meningioma, we reported 1 patient with transient third cranial nerve deficit and 1 permanent third cranial nerve deficit. One patient died as a result of intracerebral hemorrhage. Mortality for T1 and T2 incisural meningiomas was 2.85%, and the overall morbidity rate was 23% (6 patients). The average follow-up duration was 68.5 months (range = 14–308 months).

At 48 months follow-up, the patients have been clinically evaluated. None of the patients have been under worsened condition compared to the immediate postoperative time.

### Follow-Up

**Figure 3:**
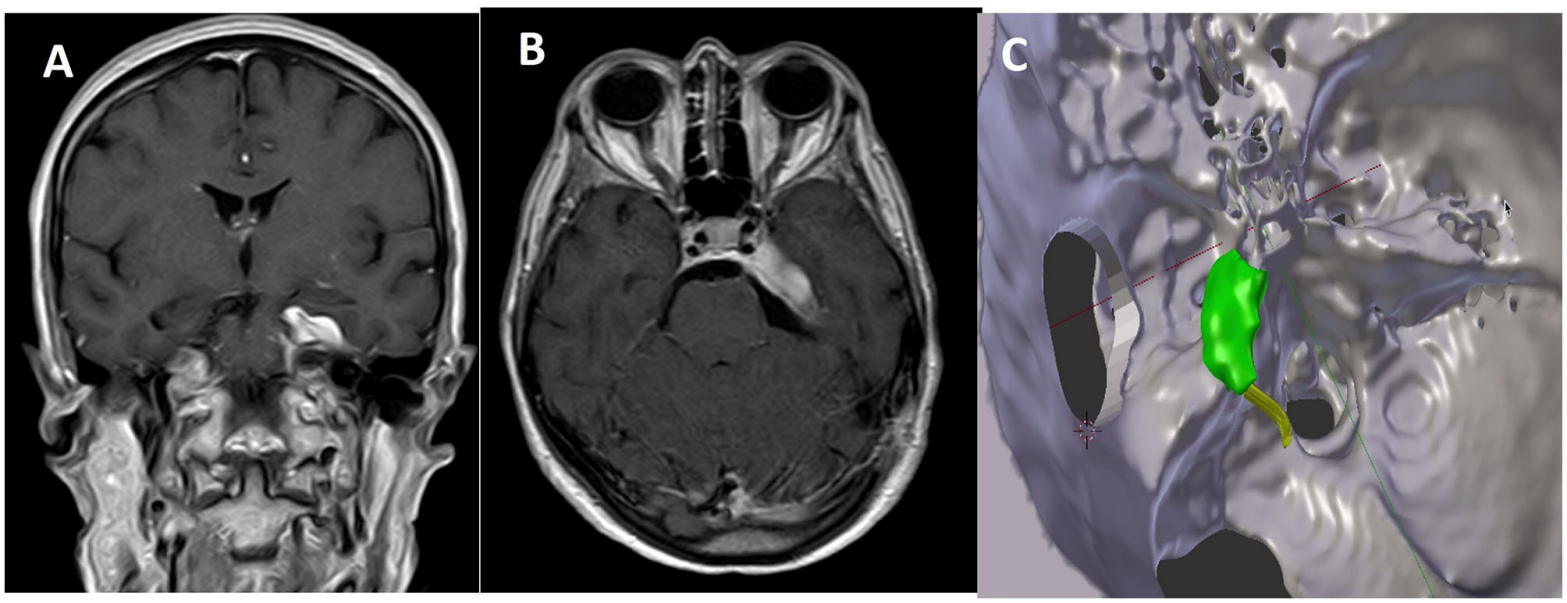
Comparison between postoperative MR in coronal **(A)** and axial **(B)** view and the expected residual processed by 3D planning **(C)**.

**Figure 4:**
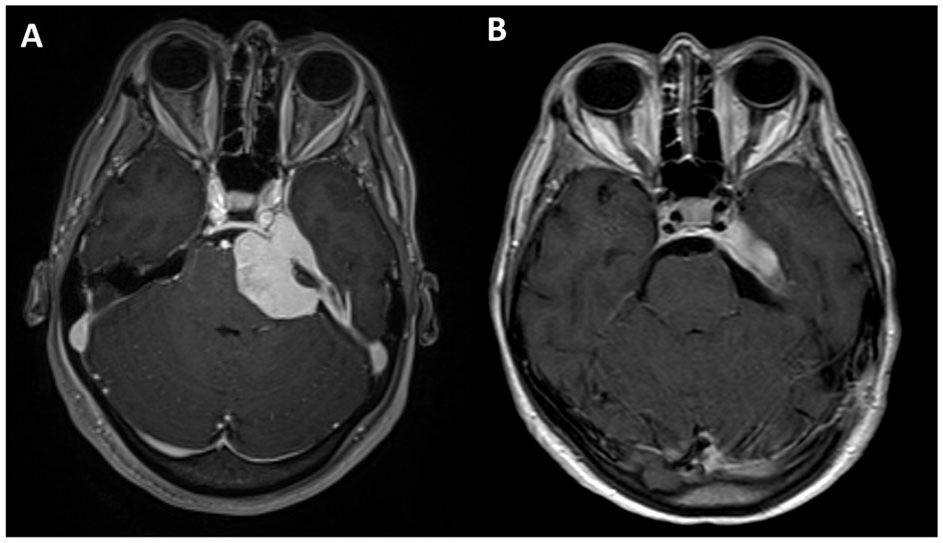
Axial view of T1-weighted MRI sequences with gadolinium: **(A)** preoperative and **(B)** postoperative.

Residual tumor was reported in 8 patients (30.7%). All patients who presented a residual tumor or persistent postoperative complications have been further discussed. After a multidisciplinary evaluation, 5 patients (19.2%) underwent a radiosurgical treatment (Gamma Knife, Elekta AB, Sweden), whereas the remnants 3 (11.4%) were included in the radiological follow-up. All 5 patients underwent the radiosurgical procedure 1 month after the microsurgical removal of the lesion. It has been decided to perform an adjuvant treatment, radiosurgery in these cases, when the meningiomas were Simpson grade >2 or with Simpson 1 characterized by WHO grade II or recurrence (Figures 3, 4).

We observed recurrence at the last 48 months follow-up in 6 patients (23%). They received the following treatment: reoperation in 1 (3.8%), and gamma knife surgery in 4 patients (15.3%), and 1 (3.8%) declined any treatment. All the other patients were still alive with no evidence of tumor recurrence. At the last follow-up, 18 patients (69.2%) had a score of 0, according to the mRS, 5 (19.2%) achieved a score of 2, 2 (7.7%) patients were classified as 3, and 1 (3.8%) patient as a 6 on the mRS.

### 3D Virtual Simulation of Tumor Resection

In 6 cases (23%), we applied a preoperative virtual process 3D reconstruction and tumor resection from 2 alternative routes. Then we compared the virtual tumor residuals. Two cases were T1 and 4 cases were T2.

The virtual images created allowed a preliminary visualization of the tumor residue, without interfering with intraoperative surgical decisions. In this latest subgroup of 6 patients, no mortality was reported. No permanent morbidity was observed. Two transitory third cranial nerves deficits were completely recovered after 3 months. The mean tumor size was 36.8 mm. According to Simpson classification, we individuated 5 patients in Simpson grade II and 1 patient in Simpson grade I. The use of virtual simulation for the surgical management of tentorial notch meningiomas allowed us to be able to better define the tumor residue with the retrosigmoid approach through the ability to define tumor exposure (Figure 1).

### Review of the Literature

The review of the literature has been summarized in Table 3. The literature search resulted in 5 original articles, which resulted in 97 patients, from 1988 to 2021. A total of 7 articles have been selected, resulting in a total of 261 patients (2, 6–11). All patients underwent microsurgical resection of their lesions. It has not been specified the rate of extent of resection (EoR) for each patient, but in all the cases, maximum safe resection was always the aim. The mortality ranged from 0 up to 9.8%. The studies did not report any correlation between the dimension and the mortality. Concerning the morbidity, it ranged from 14% up to 42.7%.

**Table 3:** Review of the literature.

**Author**	**Year**	**Total N**	**N TNM**	**Morbidity**	**Mortality**	**>3 cm**
Hashemi et al. (8)	2010	21	21	42.8%	0	
Aguiar et al. (10)	2010	30	3	23%	3%	2
Colli et al. (9)	2008	29	8	27.6% trans 13% cn deficit permanent T1-T2	0	2
Bassiouni et al. (2)	2004	81	19 T1-T2	26% permanent complications	5.3% T1-T2	
Samii et al. (6)	1996	25	25 19 T1-T2 6 T3	28%	0	39
Guidetti et al. (11)	1988	61	7	34% transient 7% permanent	9.8%	50.8% 2-5 cm−5 cm
Talacchi et al. (7)	2018	14	14	14% systemic and neurological complications	0	

## Discussion

Tentorial meningiomas are a rare subgroup of intracranial meningiomas, representing 3–6% of the total (2, 3). Moreover, they represent an anatomical and surgical unique entity. Based on Yasargil classification, we individuated T1 and T2 localization as the focus of our analysis, presenting the largest case series. Due to the rarity of this disease and the lack of data concerning incisural tentorial meningiomas, no detailed management is planned. Analyzing our data and pertinent literature report, we described an update on the management of tentorial notch meningiomas.

### Demographical Data

Reports in the literature showed that this type of lesion affects mainly middle age patients. In our series, incisural meningiomas affected the fifth decade of life, with a female/male ratio of 1.8. Hashemi reported a mean age of 56 years old, Samii a mean of 52 years, recently Talacchi et al. reported a mean age of 52.8 years (7).

Typically, we can observe a predominance in female sex in all pertinent series 17 F/4 M in Hashemi case series, and 18 women and 7 men in Samii series. Only Talacchi et al. described an equal incidence in men and women (7).

### Symptoms and Sign Onset

The most frequent symptoms described in different series were trigeminal pain (36%), headache (32%), gait disturbance (28%), hearing loss (24%), dizziness (12%), and diplopia (12%) reported by Samiiet et al. Visual defects were main symptoms described by Hashemi. The incidental discovery of such a lesion is quite rare.

The cranial deficit was the most onset neurological sign, especially the third and fifth cranial nerves (6–8, 12–14). Colli reported a high percentage of intracranial hypertension. Similarly, our series reported the main incidence of cranial nerve deficit as the neurological onset.

### Tumor Characteristic

Hashemi described a median size tumor of 25 mm (range 1–6 cm), Samii reported a tumor diameter ranging from 10 to 60 mm, Talacchi labeled a mean tumor size of 42.5 mm with a range of 25–52 mm. Our series evaluated a mean tumor size of 35 mm in a range of 26–48 mm.

According to Simpson's classification, grade I or II tumor removal was more than 80% in all series, slightly lower in the patients series of Talacchi (64.3%). We reported a Simpson grade I and II removal in 22 (84.6%) patients and histopathological examination revealed anaplastic meningioma in 2 patients (7.7%).

### Postoperative Complications

The most common postoperative complications were third nerve palsy (6.3% in Hashemi series), palsy of forth nerve (28% in Samii series), and hemiparesis 15.8% in the Bassiouni series (2, 8). Two systemic complications, pleural effusion and urinary tract infection, were documented in Talacchi's study (7). Our main complication was third cranial nerve palsy.

Incisural meningiomas are associated with a low rate of mortality, only Bassiouni reported a 5.3% of death and we reported only 1 case (3.8%).

### Recurrence

The mean length of follow-up was very long for all series, a low rate of recurrence was reported. We reported a 30.7% percentage of residual tumor and 23% of rate recurrence. In 19% of residual and 15.3% of recurrence, we suggested radiosurgery treatment.

Our results support literature evidence. Incisural meningiomas affected, principally, adults in fifth decade of life, with a sex female predominance. Tentorial notch meningiomas are symptomatic lesion, which can result in cranial nerve deficit or intracranial hypertension. The microsurgical resection of the mass is the gold standard in incisural meningiomas treatment. The objective is the gross total resection (GTR). However, achieving this goal is a challenge due to the presence of neurovascular structures in this complex anatomic area. Surgical approach selection depends on the exact site of the attachment and the size of the tumor. Moreover, it is essential to consider a connection with neurovascular structures surrounding the tumor. In spite of a low mortality rate, surgical procedure is related to a high morbidity rate. Cranial nerve deficit and hemiparesis significantly impact the patient's life. Our morbidity overall rate was 23%, It was found in patients with lesions larger than 3 cm. Sixty-one percentage of the patients in our series presented lesions >3 cm at diagnosis.

Furthermore, our data suggest that the management of tentorial meningiomas larger than 3 cm with a sole surgical treatment increases the incidence of postoperative complications due to major involvement of the neurovascular structures surrounding tumor. Anatomically, this is probably caused by the fact that larger lesions did not allow a good visualization of the clival plane. Tumor consistency plays a role, since meningiomas with soft consistency are easier to remove with a lower rate of aggression and inflammation in the tumor bed. Therefore, the aim of the microsurgical resection should be the maximal safe resection, with the fundamental preservation of the nearby neurovascular structures. Because of such technical difficulties, such cases should be discussed with a multidisciplinary team, making use of modern virtual method, intraoperative monitoring of cranial nerve, sensitive and motor functions, postoperative treatment (radiotherapy and radiosurgery).

In the modern neurosurgical era, we must consider alternative possibilities to radical surgery in order to preserve neurovascular structures adjacent to the lesions. The current results of Stereotactic radiosurgery (SRS) modified the clinical and surgical strategy. Partial debulking followed by adjuvant SRS achieves high tumor control rates with a lower level of complication (14, 15). A careful preoperative planning allows to plan the tumor residual to be subjected to gamma knife. The 3-D approach simulation allows an accurate study of the anatomical and neurovascular structure before surgery and permits a clear anatomical reconstruction to reach the best safest resection in accordance with the radiotherapist design. Wide surgical approaches should be abandoned in favor of well-planned essential surgical corridors. The craniotomy can be reduced and tailored on the residual tissue, well-planned before surgery. Preoperative virtual simulation and volumetric quantification of tumor digitally allow to evaluate different surgical approach and to define maximum resectable volume and maximum visible surface; 3D virtual simulation of tumor resection from alternative routes offers a valid support to standard preoperative decision making.

We advise a multidisciplinary approach to plan incisural meningiomas supported by 3D simulation and virtual reconstruction. Considering preoperatively a residual tumor followed by radiosurgery in order to decrease morbidity risk, especially in lesions >3 cm.

### Study Limitations

Our findings should be interpreted contemplating the limitations of the study. Despite the best efforts, the study is characterized by some limitations. The most evident is the nature itself of the study since it presents the design of a retrospective study.

Despite great promises given by this new technology, which can be applied in different fields of neurosurgery, virtual simulation for surgical resection of intracranial tumor still needed scientific validation to better define its reliability. The study design relating to the use of this virtual process does not contain systematic statistics or validation data for this method. Despite this system still lacks scientific validation, we still encourage other surgeons to integrate it into their standard decision-making process.

## Conclusion

Despite agreeing on the fundamental role of surgery for the management of tentorial notch meningiomas, the best surgical approach is still a topic of discussion. Because of the high rate of postoperative morbidity, such lesions should be managed by a team of experienced neurosurgeons. Our experience suggests that a good preoperative planning is associated with a good surgical outcome and a preserved quality of life. Tentorial Meningioma (TMs) located at the tentorial edge carried a definitely worse prognosis than peripheral forms.

The use of virtual simulation allows a reduction in postoperative morbidity and mortality, through a careful analysis of the possible tumor exposure and the possibility to define a residual tumor. Because of the higher rate of postoperative complications, neurosurgeons should not hesitate to take advantage of adjuvant therapy, like SRS, which could safely treat the remnants, avoid part of the risk of an aggressive neurosurgical removal.

## Data Availability Statement

The original contributions presented in the study are included in the article/supplementary material, further inquiries can be directed to the corresponding author.

## Ethics Statement

The studies involving human participants were reviewed and approved by IRB of the Humanitas Research Hospital (Reference n°: IRB- 1459). The patients/participants provided their written informed consent to participate in this study.

## Author Contributions

AC: conceptualization. DC, DM, and FN: data curation. DC and DM: formal analysis and visualization. VD'A: resources. FN: software. FS and MF: supervision. AC and MF: validation and writing—review and editing. AC, DC, and IZ: writing—original draft. All authors have read and agreed to the published version of the manuscript, contributed to the article, and approved the submitted version.

## Conflict of Interest

The authors declare that the research was conducted in the absence of any commercial or financial relationships that could be construed as a potential conflict of interest.

## Publisher's Note

All claims expressed in this article are solely those of the authors and do not necessarily represent those of their affiliated organizations, or those of the publisher, the editors and the reviewers. Any product that may be evaluated in this article, or claim that may be made by its manufacturer, is not guaranteed or endorsed by the publisher.
